# Therapy for SIAD: what does the future hold?

**DOI:** 10.1177/20420188231166493

**Published:** 2023-04-19

**Authors:** Ploutarchos Tzoulis

**Affiliations:** Division of Medicine, Department of Metabolism & Experimental Therapeutics, University College London Medical School, Gower Street, London WC1E6BT, UK

**Keywords:** empagliflozin, hyponatraemia, SIAD, sodium, tolvaptan, urea

Hyponatraemia, the most frequent electrolyte abnormality, is defined as serum sodium concentration below 135 mmol/l and is associated with significant morbidity and excess mortality, with its commonest cause being SIAD (syndrome of inappropriate antidiuresis). Except for patients with severe symptoms requiring treatment with hypertonic saline, the vast majority of patients with SIAD experience mild to moderate symptoms and receive fluid restriction as first-line therapy. The last major international guidelines, published in 2014, agreed on the limited evidence base of therapeutic approach, while they led to diverging recommendations with regard to the suggested second-line therapies.^1,2^ A US consensus statement suggests the use of tolvaptan as second-line agent^1^ in contrast to the European guidelines which do not recommend its use, suggesting oral urea instead.^2^ In the era of evidence-based medicine, there has been surprising sparsity of high-quality data for the treatment of SIAD until 2020, with the exception for placebo-controlled trials of vasopressin receptor antagonists.

Fluid restriction, the mainstay treatment for SIAD in the last decades, had not been prospectively evaluated until recently. Real-world data have recorded its limited efficacy, with a response rate estimated at around 50%.^3^ Fluid restriction is also unlikely to achieve significant sodium correction in cases of limited free water excretion, such as when urine osmolality exceeds 500 mOsm/kg or the serum to urine electrolyte ratio is greater than 1.^1^ In 2020, the first randomised controlled trial (RCT) of fluid restriction for SIAD was published, showing significant, but modest, efficacy, as evidenced by a median sodium rise of 3 mmol/l over a 72-h period, with one third of patients being non-responders.^4^ These data highlight the need for additional therapies in a substantial proportion of patients. The choice of second-line therapy has been a subject to great debate with options including tolvaptan, urea, combination of furosemide and sodium chloride, demeclocycline, and, recently, empagliflozin.

Tolvaptan, an oral selective arginine vasopressin antagonist, is the logical and highly attractive therapeutic option for SIAD on the basis of the underlying pathophysiological mechanism. Tolvaptan, the sole therapy approved by regulatory authorities across all continents since 2008, is highly efficacious in increasing serum sodium concentration through significant aquaresis.^5^ Real-world data have confirmed the great efficacy of tolvaptan, but they have also reported overly rapid hyponatraemia correction in a substantial proportion of patients.^6^ The lower the baseline serum sodium, the higher the risk of over-rapid correction.^5,6^ For this reason, lower than approved initial tolvaptan doses, such as 7.5 mg, are frequently used in routine clinical care with the hope of lowering the risk of overcorrection, without having been evaluated in prospective studies. The fact that tolvaptan often acts as a cannon rather than a magic bullet in combination with its high cost has limited its use in selected cases.

With regard to urea, the main alternative pharmacotherapy for SIAD, it is an osmotic agent with evidence base of low quality which is mainly used in some European countries. The other barriers to its use include the lack of an approved preparation, its limited availability and its poor palatability. The utilisation of other pharmacotherapies, such as demeclocycline and combination of furosemide and sodium chloride, is also based on small case series and is limited due to concerns about their renal safety.

Empagliflozin, routinely used as an antidiabetic medication, has been the most recent addition to the list of drug candidates for SIAD. Empagliflozin, an SGLT-2 (sodium-glucose co-transport 2) inhibitor, increases urinary water excretion in SIAD through osmotic diuresis. The first RCT of empagliflozin 25 mg per day *versus* placebo, as adjunct to fluid restriction 1000 ml per day, showed a significantly higher serum sodium increase by 3 mmol/l over a 4-day period without any adverse events.^7^ A subsequent RCT of 4-week monotherapy with empagliflozin *versus* placebo showed promising results, with a corresponding median serum sodium increase of 4.1 mmol/l.^8^ The advantages of empagliflozin are its wide availability and broad clinical experience as well as its well-established safety profile with long-term cardiovascular and nephroprotective effects, but the effect on sodium rise, if any, seems to be modest.

The key, still unanswered, question remains whether correction of hyponatraemia will translate into improvement of hard, clinically meaningful, patient-related outcomes. An ongoing large RCT will hopefully answer the long-standing question whether correction of inpatient hyponatraemia can improve mortality and reduce readmission rate.^9^ In the outpatient setting, large prospective studies are needed to test the hypothesis that sodium normalisation can improve neurocognition, quality of life, and, potentially, bone mineral density and fracture rate. The justification for widespread use and reimbursement of pharmacotherapies for SIAD will finally be determined by their impact on hard patient-related outcomes and results of head-to-head studies, comparing various treatment options.

What will the future for SIAD treatment hold? The low efficacy and difficult adherence to fluid restriction highlight the need to expand the use of pharmacotherapy in clinical practice. Vaptans are the main candidates for widespread use, provided that we will have access to dosing and titration regimens, combining high efficacy and low risk of overly rapid correction at an affordable cost. Besides vaptans, SGLT-2 inhibitors will probably have a role as an adjunct treatment for chronic mild hyponatraemia.

## Declarations

**Ethics approval and consent to participate:** Not applicable.

**Consent for publication:** Not applicable.

**Availability of data and materials:** Not applicable.

## References

[1] Verbalis JG Goldsmith SR Greenberg A , et al. Diagnosis, evaluation, and treatment of hyponatremia: expert panel recommendations. Am J Med 2013; 126(Suppl. 1): S1–S42. 10.1016/j.amjmed.2013.07.006 24074529

[2] Spasovski G Vanholder R Allolio B , et al. Clinical practice guideline on diagnosis and treatment of hyponatraemia. Eur J Endocrinol 2014; 170: G1–G47. 10.1530/EJE-13-1020 24569125

[3] Verbalis JG Greenberg A Burst V , et al. Diagnosing and treating the syndrome of inappropriate antidiuretic hormone secretion. Am J Med 2016; 129: 537.e9–537.e23. 10.1016/j.amjmed.2015.11.005 26584969

[4] Garrahy A Galloway I Hannon AM , et al. Fluid restriction therapy for chronic SIAD; results of a prospective randomized controlled trial. J Clin Endocrinol Metab 2020; 105: dgaa619. 10.1210/clinem/dgaa619 32879954

[5] Schrier RW Gross P Gheorghiade M , et al. Tolvaptan, a selective oral vasopressin V2-receptor antagonist, for hyponatremia. N Engl J Med 2006; 355: 2099–2112. 17105757 10.1056/NEJMoa065181

[6] Tzoulis P Waung JA Bagkeris E , et al. Real-life experience of tolvaptan use in the treatment of severe hyponatraemia due to syndrome of inappropriate antidiuretic hormone secretion. Clin Endocrinol 2016; 84: 620–626. 10.1111/cen.12943 26385871

[7] Refardt J Imber C Sailer CO , et al. A randomized trial of empagliflozin to increase plasma sodium levels in patients with the syndrome of inappropriate antidiuresis. J Am Soc Nephrol 2020; 31: 615–624. 32019783 10.1681/ASN.2019090944 PMC7062212

[8] Refardt J Imber C Nobbenhuis R , et al. Treatment effect of the SGLT2 inhibitor empagliflozin on chronic syndrome of inappropriate antidiuresis: results of a randomized, double-blind, placebo-controlled, crossover trial. J Am Soc Nephrol 2023; 34: 322–332. 36396331 10.1681/ASN.2022050623 PMC10103093

[9] Refardt J Pelouto A Potasso L , et al. Hyponatremia intervention trial (HIT): study protocol of a randomized, controlled, parallel-group trial with blinded outcome assessment. Front Med 2021; 8: 729545. 10.3389/fmed.2021.729545 PMC8450416 34552947

